# Criteria for judging the immune markers of COVID‐19 disease vaccines

**DOI:** 10.1002/mco2.109

**Published:** 2021-12-31

**Authors:** Nan Lin, Haoxuan Fu, Dan Pu, Yuxin Quan, Yueyi Li, Xiaomeng Yin, Yuhao Wei, Hang Wang, Xuelei Ma, Xiawei Wei

**Affiliations:** ^1^ West China School of Medicine West China Hospital Sichuan University Chengdu China; ^2^ Department of Statistics University of Illinois at Urbana Champaign Urbana Illinois USA; ^3^ Department of Radiation Oncology Cancer Center, West China Hospital, Sichuan University Chengdu China; ^4^ Department of Biotherapy Cancer Center, West China Hospital, Sichuan University Chengdu China; ^5^ Laboratory of Aging Research and Nanotoxicology, State Key Laboratory of Biotherapy National Clinical Research Center for Geriatrics, West China Hospital, Sichuan University Chengdu China

**Keywords:** assessment, neutralizing antibodies, SARS‐CoV‐2, vaccine

## Abstract

As severe acute respiratory syndrome coronavirus 2 (SARS‐CoV‐2) sweeping the world, effective and affordable vaccines are in urgent need. A reliable system for the assessment of SARS‐CoV‐2 vaccines would boost the development of vaccines and reduce the research cost. We constructed a logistic regression model and analyzed the relationship between antibody (Ab) level and efficacy of different vaccine types. The relationship between assessment dates and Ab levels was depicted by plotting the mean of Ab levels evolved over time and a fitted cubic polynomial model. Anti‐spike immunoglobulin G (IgG) could best estimate the vaccine efficacy (VE) (adjusted *R* ^2^^ ^= 0.731) and neutralizing Ab to live SARS‐CoV‐2 also explained a fine relationship (adjusted *R* ^2 ^= 0.577). Neutralizing Abs to live SARS‐CoV‐2 in inactivated virus vaccines reached a peak during days 40–60, and their receptor‐binding domain (RBD)‐IgG peaked during days 40–50. For messenger RNA (mRNA) and viral vector vaccines, their neutralizing Ab to live SARS‐CoV‐2 peaked later than day 40, and for RBD‐IgG during days 30–50. For mRNA and viral vector vaccines, their peak time of Abs was later than that in inactivated virus vaccines. RBD‐IgG peaked earlier than Ab to live SARS‐CoV‐2. Anti‐spike IgG and Ab to live SARS‐CoV‐2 may be good immune markers for VE assessment.

## Graphical abstract

We evaluated different Abs and assessment times for coronavirus disease vaccines. We found anti‐spike immunoglobulin G (IgG) may best estimate the vaccine efficacy and neutralizing Ab to live severe acute respiratory syndrome coronavirus 2 (SARS‐CoV‐2) also explained a fine relationship. The peak time of different Abs in messenger RNA virus was later than that in inactivated virus vaccines, and receptor‐binding domain‐IgG peaked earlier than Ab to live SARS‐CoV‐2.

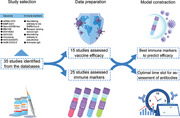

## 1 INTRODUCTION

With approximately 250 million infections and five million deaths worldwide, more than 320 coronavirus disease 2019 (COVID‐19) vaccines were in development according to the World Health Organization (WHO) on 19th November, 2021.^1^ Multiple types of vaccines showed varied vaccine efficacies (VEs) ranging from 50% to 95% against symptomatic COVID‐19 infections in phase III clinical studies; seven of them have been approved for marketing or emergency use by WHO.^2^, ^3^, ^4^, ^5^, ^6^, ^7^, ^8^, ^9^, ^10^, ^11^, ^12^, ^13^, ^14^ Approved vaccines are mainly classified into four types: inactivated virus vaccines like CoronaVac, BIBP‐CorV, and BBV‐152; adenoviral vector vaccines like Ad26.COIV2.S; protein subunit vaccines like ChAdOx1‐S; and mRNA encoding vital proteins like mRNA‐1273 and BNT162b2. According to the characteristics of these vaccines, the safety and efficacy might be different. Until now, several meta‐analyses have summarized the efficacy of COVID‐19 vaccines, which showed mRNA and adenoviral vector vaccines have greater VE than inactivated virus vaccines and protein‐based vaccines.^15^, ^16^, ^17^, ^18^ With the manufacturing challenges caused by the global demand, more affordable vaccines with good safety and efficacy were in urgent need.^19^, ^20^ Therefore, it became essential to identify proper biomarkers to effectively evaluate the efficacy of developing and developed vaccines.

The protection rate of a developing COVID‐19 vaccine was assessed by a phase III clinical study with at least 1000 participants.^21^ Before the phase III study, antibodies (Abs) like neutralizing Abs to live severe acute respiratory syndrome coronavirus 2 (SARS‐CoV‐2), receptor‐binding domain (RBD)‐IgG, anti‐spike immunoglobulin G (IgG), and binding Ab were collected in the phase I/II studies.^22^, ^23^, ^24^ Although SARS‐CoV‐2‐specific memory T‐cell immunity has shown a certain degree of protection, more evidence of the relationship between immune biomarkers and protection rate exists.^25^, ^26^, ^27^ Evidence from both animal and human studies have shown that a higher level of the pre‐existing neutralizing Abs is potentially related to protection.^28^, ^29^ Study has demonstrated that higher peri‐infection neutralizing Ab titers were associated with lower viral load, which is related to infectivity.^30^, ^31^ Studies based on mRNA‐1273 and ChAdOx1‐S have also been conducted in order to predict the relationship between Ab titers and protection rate.^32^, ^33^ These studies confirmed that neutralizing Abs against live virus seems a great predictor for the protection rate of a promising COVID‐19 vaccine. Yet, more Abs like RBD‐IgG and anti‐spike IgG have not evaluated their association between protection. As is well‐known, spike protein is one of the four structural proteins of the pericapsid of SARS‐CoV‐2, which are the main immunogen. Spike protein plays an important role in viral binding, fusion, and replication within host cells by interacting with the angiotensin conversion enzyme human 2. RBD is a domain of the S1 subunit of spike protein that interacts directly with the receptors of the host cells. Therefore, the emerging of RBD‐IgG and anti‐spike IgG might indicate the ability to prevent pathogens from entering the host cells.

The assessment time and method vary during different phase I/II studies.^34^, ^35^, ^36^ Even though many studies chose the enzyme‐linked immunoassay method and days 0, 28, 42, and 56 to evaluate Abs, few studies explained when the Abs can reach their highest concentration. Studies found that for the BNT162b2 vaccine, the Abs reached the peak during days 4–30 after receipt of the second dose of vaccine.^37^ Evidence from the real world also showed that for two doses of BNT162b2, anti‐spike IgG levels peaked 28 days after the first vaccination, regardless of age.^38^ However, for different immune markers and different types of vaccines, no general guidance has been developed for when and how to evaluate the Abs.

How to judge the immune markers was an essential question for evaluating the VEs. To the best of our knowledge, no comprehensive study has been conducted to solve those questions generally. In this regard, we conducted an analysis based on the previous phase I–III clinical researches of COVID‐19 vaccines. In this study, we summarized and modeled the immune markers and VE for different vaccines, which identified Abs that were mostly related to the protection rate, and also identified the best time to assess the Abs for different vaccines, thus providing advice for future vaccine research and development.

## 2 RESULTS

### 2.1 Characteristics of included studies

**FIGURE 1:**
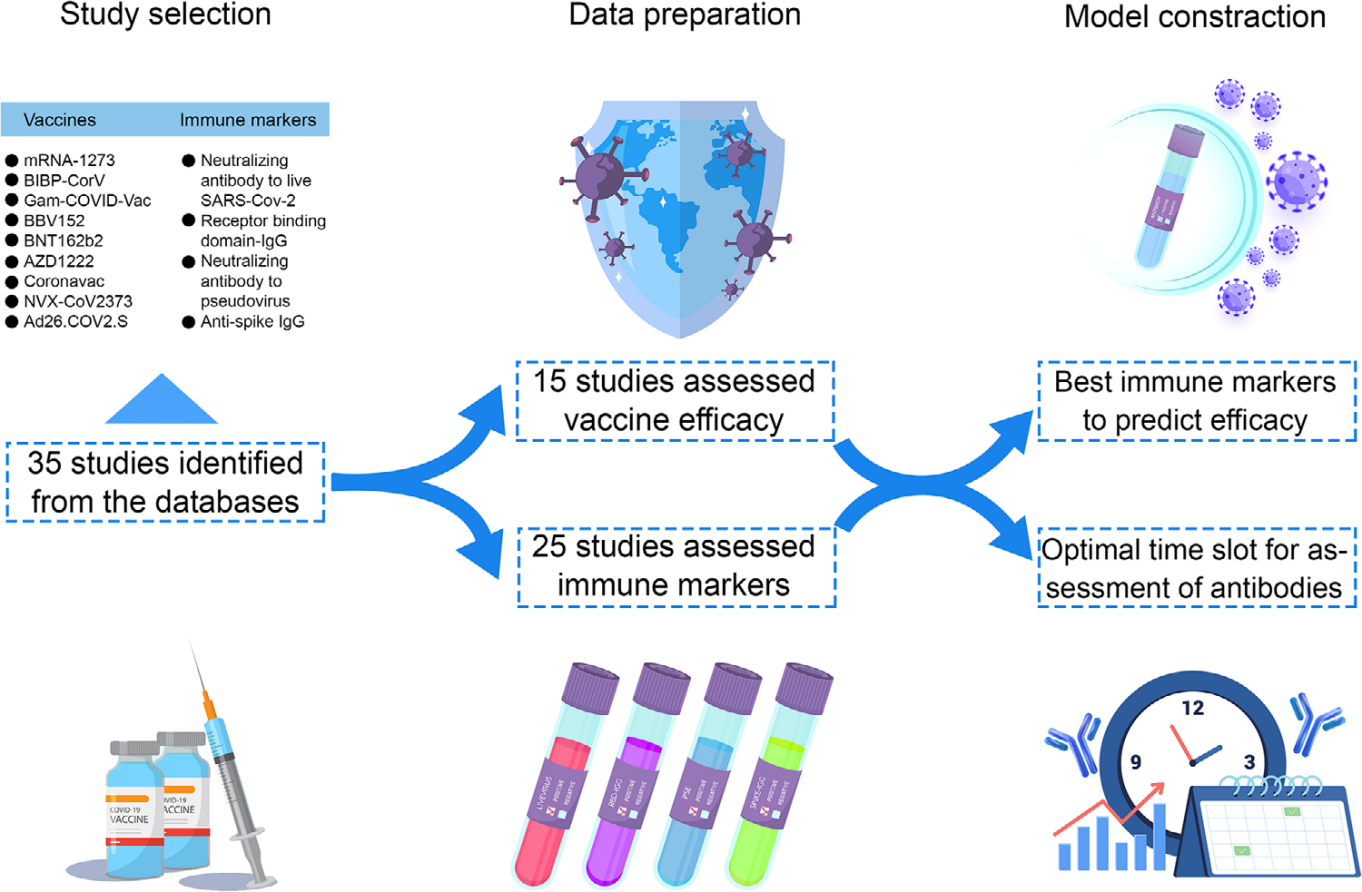
The flowchart of the study

The flowchart of the study was shown (Figure 1). After searching from databases, a total of 35 studies were finally included in this study with 15 phase III studies for nine vaccines (Figure S1 and Table S1). Nine vaccines were included, with two mRNA vaccines (mRNA‐1273 and BNT162b2), three virus‐vectored vaccines (ChAdOx1‐S, Ad26.COV2.S, and Gam‐COVID‐Vac), three inactivated vaccines (CoronaVac, BIBP‐CorV, and Covaxin), and one protein subunit vaccine (NVX‐CoV2373). In order to compare the assessment methods of different vaccines, we also summarized these methods in different studies (Table S2), including the dose, type of the Ab, details of the method, schedule, and assessment time.

### 2.2 Superiority of anti‐spike IgG and neutralizing Ab to live SARS‐CoV‐2

**FIGURE 2:**
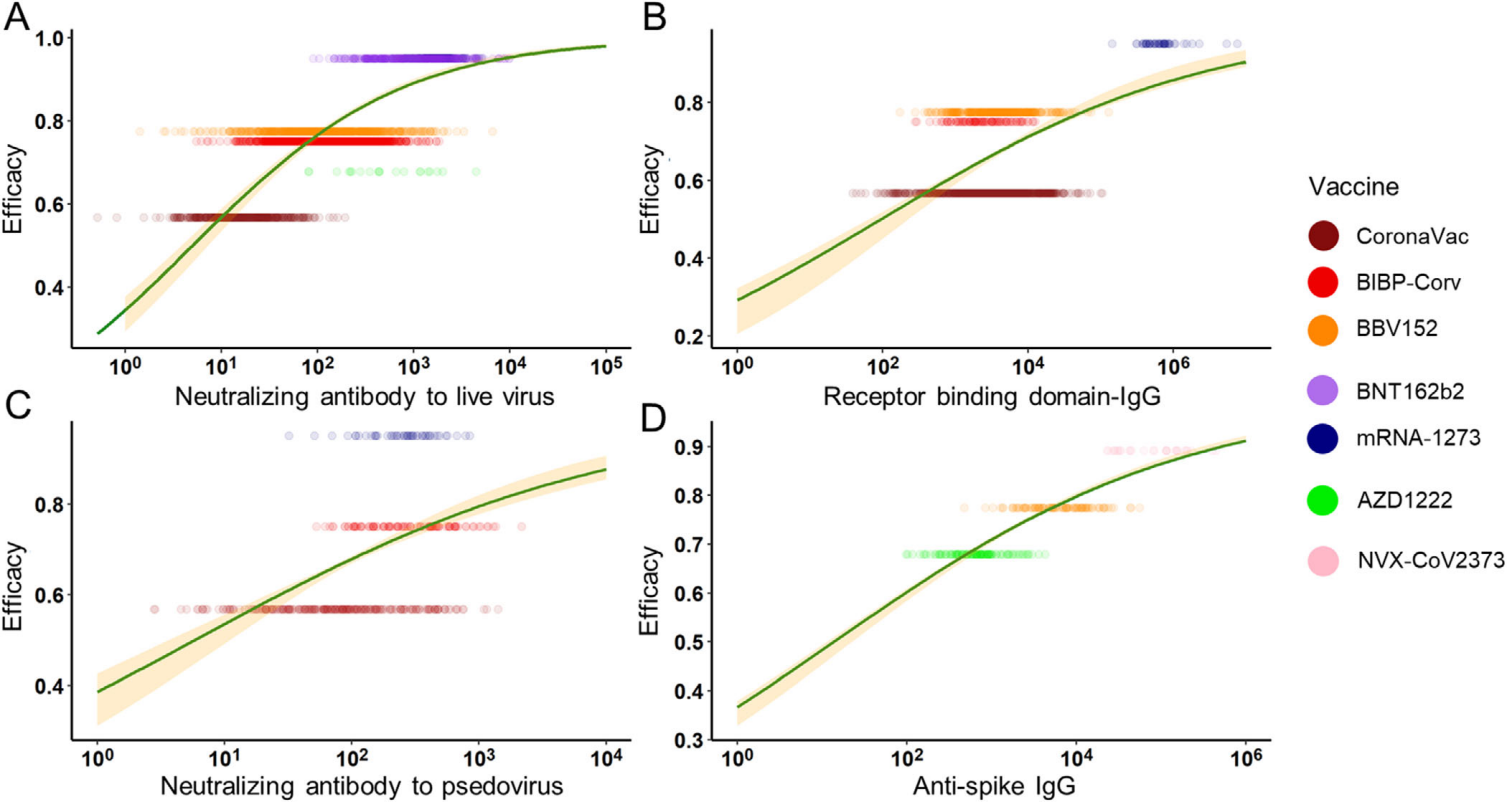
The relationship between antibodies and vaccine efficacy. In the general population, the prediction power of vaccine‐induced antibodies was ranked as anti‐spike immunoglobulin G (IgG) (D) (adjusted *R* ^2^ = 0.731), neutralizing antibody (Ab) to live severe acute respiratory syndrome coronavirus 2 (SARS‐CoV‐2) (A) (adjusted *R* ^2^ = 0.577), receptor binding domain‐IgG (B) (adjusted *R* ^2^ = 0.264) and neutralizing antibody to pseudovirus (C) (adjusted *R* ^2^ = 0.153)

According to our method, the geometric mean titer (GMT) and standard deviation (on a log_10_ scale) were collected and calculated in the included studies to compare different Abs. When two assessment methods were used in one study, we chose the method most commonly used in order to reduce the bias. We also generated the combined VE using the pre‐set formula (Table S3). Finally, we selected seven vaccines with enough data to be modeled. Among these, six vaccines were assessed for neutralizing Ab to live SARS‐CoV‐2, four vaccines for RBD‐IgG, three vaccines for neutralizing Ab to pseudovirus (PSE), and three vaccines for anti‐spike IgG. To compare different Abs used for vaccine assessment, we used logistic regression to fit the relationship between efficacy and vaccine‐induced Ab levels. Adjusted R^2^, the level of total variation explained by the model after adjusted for model complexity, was used to judge the performance of the immune markers. In the general population, the model showed that the anti‐spike IgG level best explained the relationship with protection across the studies (adjusted *R* ^2 ^= 0.731). The neutralizing Ab to live SARS‐CoV‐2 also explained a fine relationship with protection (adjusted *R* ^2 ^= 0.577), while for RBD‐IgG and PSE, the relationship between protection was inferior (adjusted *R* ^2 ^= 0.264 and 0.153) (Figure 2). For these models, studies were excluded due to the lack of essential information about Abs or they stood out as outliners.

**FIGURE 3:**
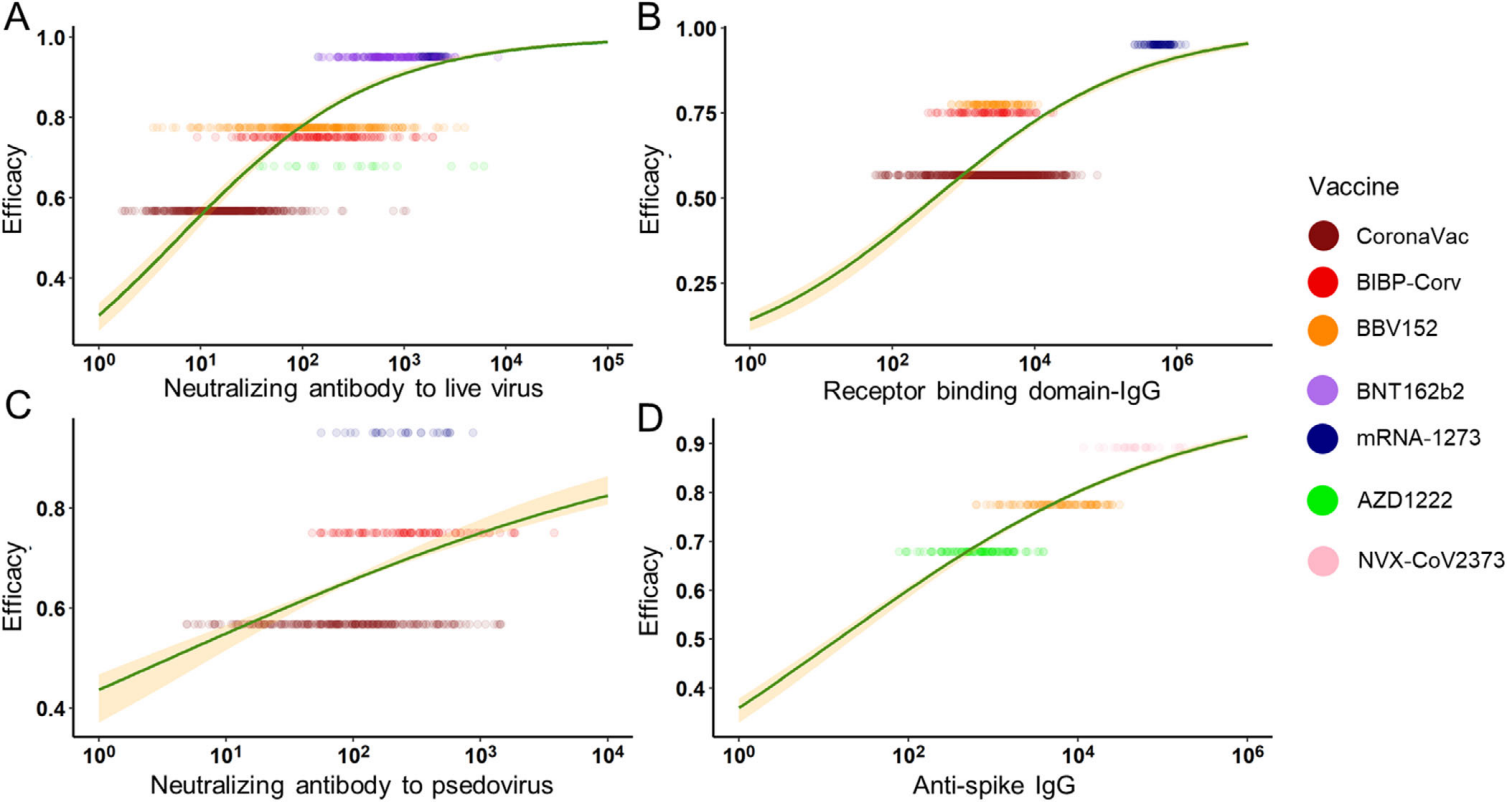
The relationship between antibodies and vaccine efficacy in 16–65 years subgroup. In the subgroup, the prediction power of vaccine‐induced antibodies was ranked as anti‐spike immunoglobulin G (IgG) (D) (adjusted *R* ^2^ = 0.694), neutralizing antibody (Ab) to live severe acute respiratory syndrome coronavirus 2 (SARS‐CoV‐2) (A) (adjusted *R* ^2^ = 0.577), receptor binding domain‐IgG (B) (adjusted *R* ^2^ = 0.484) and neutralizing antibody to pseudovirus (C) (adjusted *R* ^2^ = 0.115)

Moreover, to reduce the possible bias caused by different age groups, we chose the age range of 16–65 years for the subgroup study. In the subgroup analysis, neutralizing Ab to live SARS‐CoV‐2 and RBD‐IgG showed a stronger correlation with protection (adjusted *R* ^2 ^= 0.577 and 0.484), while anti‐PSE and anti‐spike IgG still maintained (adjusted *R* ^2 ^= 0.115 and 0.694) (Figure 3). The improvement of adjusted *R* ^2^ was mainly caused by the decline of the standard deviation in different vaccines. In conclusion, both in the general population and 16–65 years’ subgroup, anti‐spike IgG best explained the relationship with protection across the studies. The neutralizing Ab to live SARS‐CoV‐2 performed fine explaining the relationship with protection. RBD‐IgG may be a good predictor, but its performance may be greatly affected by age. The PSE was not recommended to be an indicator for protection.

### 2.3 Optimal time slot for assessment of Abs

**FIGURE 4:**
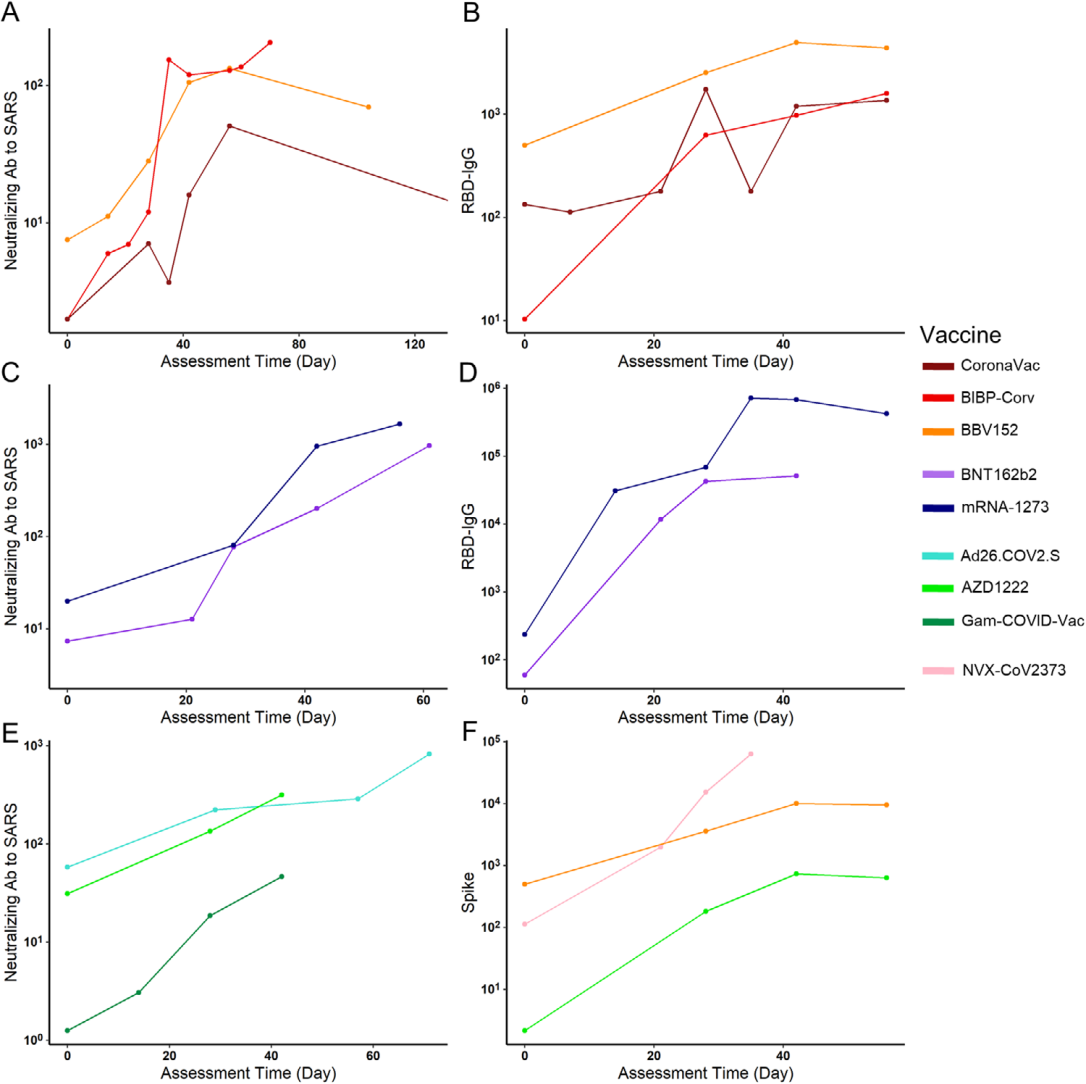
The antibody level changes of different vaccines through time after vaccination. The neutralizing antibody of live severe acute respiratory syndrome coronavirus 2 (SARS‐CoV‐2) in three inactivated viral vaccines remained at a relatively high level during 40–70 days after vaccination (A). The receptor‐binding domain (RBD)‐IgG in these three vaccines reached the highest level at about 40 days, then remained stable until about day 60 (B). For two messenger RNA (mRNA) vaccines (mRNA‐1273 and BNT‐162b2), both the neutralizing antibody to live SARS‐CoV‐2 and RBD‐IgG peaked around day 40 (C and D). Until 40 days after vaccination, neutralizing antibody to live SARS‐CoV‐2 in adenoviral vector vaccines remained increasing and did not reach the peak (E). Three vaccines with spike protein antibody results available indicated the peak time at day 40, while NVX‐CoV2373, a kind of spike protein vaccine showed an extremely high level of spike protein antibody (F)

Almost all of the included vaccines in the analysis required a second dose, except for Ad26.COV2.S. The schedule of the second injection varied, such as day 14 (CoronaVac and BBV152), day 21 (BNT162b2, Gam‐COVID‐Vac, NVX‐CoV2373, and BIBP‐CorV), day 28 (AZD1222 and mRNA‐1273). Since many vaccines only assessed their Ab levels at less than five time slots, we summarized the log_10_GMT each time slot for different vaccines and chose a line chart to describe the tendency of vaccine immune markers over time for qualitative analysis. For inactivated virus vaccines, their neutralizing Abs to live SARS‐CoV‐2 reached the peak on 40–60 days and further reached a relative plateau. The mRNA vaccines maintained an increase after 60 days and the viral vector vaccines also maintained an increase after 40 days (Figure 4A,C,E). Therefore, the assessment period of neutralizing Abs for mRNA and viral vector vaccines should be scheduled wider to get a peak concentration to guide subsequent phase III clinical trials. For inactivated virus vaccines, their RBD‐IgG peaked during days 40–50, and those in mRNA vaccines peaked during days 30–50 (Figure 4B,D). Their trends suggested that following clinical studies should select between day 30 and day 50 as the assessment time for the peak. The anti‐spike IgG was only assessed for three kinds of vaccines. It suggested that protein subunit vaccines caused a strong Ab response, but as the peak did not appear prior to day 40, an assessment date of over 40 was considered. For both viral vector vaccines and inactivated vaccines, peaks were reached during days 40–50 (Figure 4F).

**FIGURE 5:**
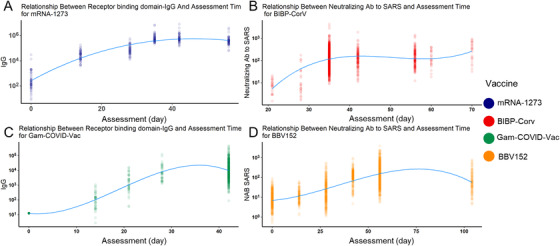
Fitted cubic polynomial models of antibody titer changes after vaccination through time that represent the relationship between antibody titer and assessment time. The receptor binding domains‐IgG reached the peak at about 35–40 days after vaccination in messenger RNA (mRNA)‐1273 severe acute respiratory syndrome coronavirus 2 (SARS‐CoV‐2) vaccine (A) and Gam‐COVID‐Vac (C). The neutralizing antibody (Ab) to live SARS‐CoV‐2 experienced a slight increase during a relatively long time, reached and maintained a high level during days 40–70 in inactivated SARS‐CoV‐2 vaccines BIBP‐CorV (B), BBV‐152 (D), which was in accordance with the reality showed in Figure 3

We also modeled the relationship between different Abs and assessment times to better understand this question. Four vaccines were chosen because they have at least five assessment time slots. Similar to the qualitative analysis results, the RBD‐IgG of mRNA‐1273 reached a peak around day 35 (Figure 5A). Similarly, the RBD‐IgG of Gam‐COVID‐Vac also peaked around day 35 (Figure 5C). The other two inactivated vaccines showed a different trend: for BIBP‐CorV vaccines, their neutralizing Ab to live COVID‐19 peaked during days 35–50, and BBV152 peaked after day 50 (Figure 5B,D). Besides, they both reached and maintained a high level during days 40–70. Conclusively, for mRNA and viral vector vaccines, their peak time of different Abs was later than that in inactivated virus vaccines, and RBD‐IgG peaked earlier than Ab to live SARS‐CoV‐2.

## 3 DISCUSSION

As the urgent demand for COVID‐19 vaccines, several kinds of vaccines have been approved and hundreds of vaccines are in development. The current evaluation of vaccines depends on the protection rate under phase III clinical trials, which is time‐consuming and expensive. It might be difficult for small companies, low‐income countries, and middle‐income countries to conduct such phase III clinical studies. Using immune markers more correlated with VE and a better schedule for evaluation in early studies have important implications for subsequent vaccine development. Remarkably, the US Food and Drug Administration did not recommend Abs testing to evaluate the immune response and protection efficacy after vaccination,^39^ which led to only a small number of studies so far assessing the protective effect of Ab after vaccination months later.^37^ However, as COVID ‐19 vaccines became more deeply understood, the assessment time and method became more important for promising vaccines, such as booster or cross‐species vaccination,^40^, ^41^, ^42^ thus Ab levels should also be assessed months later.

Former studies in common viruses, such as hepatitis B, poliomyelitis, and flu virus, had proven that protective Abs titers were related to VE and the models for protective duration had been well established.^43^, ^44^ Therefore, theoretically, speaking, people who generated Abs to SARS‐CoV‐2 are protected. A real‐world study in England showed that Ab‐positive health‐care workers had an 84% lower risk of infection than Ab‐negative ones^45^ and the medium protection time was 7 months. Former researches suggested that Abs are positively correlated with COVID‐19 vaccines, especially neutralizing and binding Abs.^46^, ^47^, ^48^ Although neutralizing Ab was the most prevalent marker tested in clinical trials, it is still unclear that whether neutralizing Ab titer is the best immune marker correlated with protection. In this study, we summarized the published clinical researches about SARS‐CoV‐2 vaccines and analyzed the correlations between efficacy and different Abs. We surprisingly found that anti‐spike IgG titer best described the protection rate, yet it is worth noting that only three kinds of vaccines had been tested for anti‐spike IgG in clinical trials and two of them specifically target spike protein,^11^, ^22^, ^35^ the other is an inactivated viral vaccine.^49^ It is hard to tell whether the protein subunit vaccines target spike protein‐induced higher anti‐spike IgG, and further confirmation is limited by the inaccessible published original data. Meanwhile, neutralizing Ab titer also showed a plausible relationship with protection rate in a larger range of vaccines. Seven kinds of vaccines had been tested for neutralizing Ab after vaccination and six of them were included.^50^, ^51^, ^52^, ^53^, ^54^, ^55^ Gam‐COVID‐Vac, a recombinant adenovirus‐based vaccine produced by Russia^56^ was excluded because it stood out as an outliner in our model which presented lower Abs levels with thick‐tail distribution and an overestimated efficacy. Its recent phase III clinical trials demonstrated the efficacy of 91.6% in preventing symptomatic COVID‐19 and 100% in preventing severe infection.^7^ Among the other vaccines included, neutralizing Ab titer correlated to the protection rate as that the higher the neutralizing Ab the higher the protection rate was, which is consistent with other studies.^47^ The analysis of other immune markers like RBD‐IgG and PSE did not show satisfying goodness of fit in the whole population, but in the 16–65 years population, RBD‐IgG titer showed a fine correlation with the protection rate. In other words, our study suggests that anti‐spike IgG, neutralizing Ab, and RBD‐IgG could predict the protection rate, while the performance of RBD‐IgG could fluctuate greatly based on the age group. However, PSE is not recommended in any circumstance.

Specifically, our analysis showed that mRNA vaccines (BNT162b2 and mRNA‐1273) induced the strongest neutralizing Ab and RBD‐IgG response, concordantly providing the highest protection. Inactivated vaccines seem to be inefficient in generating Abs and providing protection (Figure 2). Neutralizing Ab could prevent SARS‐CoV‐2 from invading into cells while binding Abs would bind to the virus alert immune system to destroy pathogens. mRNA vaccines showed superiority in both aspects. By now, we have modeled the relationship between Abs after vaccination with protection rate after logistic transformation with an approximately linear relationship. Researchers thus could use Abs titer to predict the efficacy mean of developing vaccines.^57^ However, it is noteworthy that immunocompromised individuals, like patients with cancer, HIV, or organ transplant, may not have detectable Abs after vaccination.^58^, ^59^, ^60^, ^61^ We did not specifically analyze the situation when humoral immunity was suppressed, but some researchers pointed out that cellular immunity still worked for those individuals. The animal experiment demonstrated that cellular immunity, including CD8+ T cells, protected convalescent macaques with low‐level Ab titer from the reinfection of SARS‐CoV‐2.^62^ It can also explain why we exclude the protein vaccines when modeling the relationship between neutralizing Ab to live virus and protection rate. They can produce a very high concentration of Abs, but due to the lack the productivity of cellular immunity, their protection capability could be quite limited.^19^, ^22^ Protein subunit vaccines contain fragments of protein from the structure of the pathogen. As a non‐endogenous antigen, protein subunit vaccine cannot be submitted through MHC‐I, so its cellular immunogenicity is relatively weak.^63^, ^64^ Thus, it is usually delivered along with adjuvants to boost immune response.

There are several limitations of this research. First, this study was based on other original researches that contain incomplete data. Second, vaccines included in our research mainly targeted subtype B.1.1.7, while over time, new virus subtypes emerged and people could get reinfected, which would correspondingly lead to a decline of protection rate and bias of phase III clinical trials. Third, even though enzyme‐linked immunoassay was applied in most trials, there were still other assays conducted, which might lead to discrepant titer levels. However, this research also has several strengths. First, the logistic model used for efficacy prediction, along with its corresponding confidence interval (CI), did not depend on distributional assumption over efficacy. Second, throughout this research, we compared different types of vaccine‐induced Abs and evaluated their capability in explaining VE. Third, we analyzed how Abs levels evolved over time for different types of vaccines. Compared with clinical researches focusing only on a single type of vaccine, our research is more instructive and comprehensive.

As COVID‐19 vaccines are being developed ceaselessly, the evaluation of their effectiveness should gradually become clear and specific. In this study, we focused on the criteria for judging the immune markers of COVID‐19 vaccines. Different from other studies,^47^ we found that anti‐spike IgG may be a more predictive immune marker than neutralizing Ab to live COVID‐19 virus with our logistic regression model. And we also analyzed the predictive performance of other immune markers in different age groups. Furthermore, we described the trends of different Abs for different vaccines over time. For mRNA and viral vector vaccines, their peak time of different Abs was later than that of inactivated virus vaccines. For future studies focusing on vaccine evaluation, this research would provide some guidance for choosing immune markers and assessment time.

## 4 METHODS

### 4.1 Regression analysis

The edge of using the Monte Carlo method to regenerate data is that it can take the number of observations in researches into account. By doing so, the regression result would fit more on those with a larger number of observations, whose statistics are therefore more reliable. Since the VE can only be between 0 and 1, the relationship between vaccine‐induced Ab level and efficacy is modeled using logistic regression:

$$\mathrm{VE}\;(\%)\;=\frac{1}{1+e^{-f\left(titer\right)}},$$

where *f*(titer) represents a linear combination of titer level and possibly its moments. Notice that since all phase III vaccines have efficacies higher than 0.5, it is convinced that the resulting models are generally unreliable regarding those with a low level of vaccine‐induced Ab, while models reveal the possible relationship between efficacy and vaccine‐induced Ab level as long as it is high. Ideally, the relationship between efficacy and vaccine‐induced Ab levels should be smooth and linear (after the logistic transformation of efficacy), therefore, generally, the model chosen for all vaccine‐induced Abs is with the following form:

$$\mathrm{VE}\;(\%)\;=\frac{1}{1+e^{-x\beta }},$$

where *x* = $\left(\begin{array}{c} 1\\ titer \end{array}\right)$ and *β* = $\left(\begin{array}{c} \beta_{0}\\ \beta_{1} \end{array}\right)$.

Such a simple linear relationship was chosen for two reasons. First, as a good estimator for VE, the relationship between the predictor and target must be simple and clear enough, that is, the relationship should be consistent and smooth, without too much curvature fluctuation along the line. Second, as the types of vaccines were now limited, applying complicated models to fit the data would incur the high risk of overfitting.

In order to evaluate the performance of different types of vaccine‐induced Ab on determining the VE, it was essential to estimate how much total variation of the data could be explained by the model. Therefore, naturally, we could use adjusted *R* ^2^ to determine the performance of the vaccine‐induced Ab. *R* ^2^ is defined as:

$$R^{2}=\frac{\sum_{i=1}^{n}{\left(f_{i}-\bar{y}\right)}^{2}}{\sum_{i=1}^{n}{\left(y_{i}-\bar{y}\right)}^{2}},$$

Furthermore, adjusted *R* ^2^ is defined based on *R* ^2^ as:

$$\text{adjusted}\;\;R^{2}=\;1-\frac{\left(1-R^{2}\right)\left(n-1\right)}{n-k-1},$$

where *n* is the number of observations and *k* is the number of predictors. Adjusted *R* ^2^, instead of *R* ^2^, was chosen because different models used to fit the data may have a different number of predictors. With adjusted *R* ^2^ as the criteria for judging vaccine‐induced Abs, the use of a larger model would be penalized, therefore avoiding overfitting. Even though the number of predictors was identical for different types of vaccines in this research, adjusted *R* ^2^ was yet a more general and acceptable approach for model comparison.

### 4.2 Bootstrap

Traditional CI estimation of linear regression is limited here as it relies on the assumption that the noise follows the normal distribution, which may not hold with respect to the VE. Therefore, instead, we used bootstrap as an alternative for CI estimation. From the Monte Carlo simulated data, we conducted random sampling on each vaccine group, with the number of sampling proportional to the number of observations in each vaccine group, with a total sampling size of 200 for each simulation. This sampling approach ensured that different researches with different numbers of observations could be appropriately weighted. Such resampling would be repeated 1000 times to construct the CI for the entire curve, that was, computing the empirical 2.5th and 97.5th quantile of efficacy for different vaccine‐induced Ab levels.

### 4.3 Vaccine‐induced Ab levels and assessment time

Vaccines were assessed at different assessment times. Therefore, at different time slots, there were different vaccine‐induced Ab levels. Though the day which generated the highest vaccine‐induced Ab level is chosen for constructing the relationship between vaccine‐induced Ab levels and efficacy, it was feasible to establish the relationship between assessment time and vaccine‐induced Ab levels for different types of vaccines. The relationship between time and vaccine‐induced Ab levels was depicted by a chart showing how vaccine‐induced Ab mean levels evolve over time.

Since for all types of COVID‐19, theoretically speaking, the vaccine‐induced Ab level will decrease after a specific assessment time, we chose to fit such relationship with the following model:

$$\begin{array}{ccc} & & \mathrm{lo}g_{10}\left(Vaccine-Induced\;antibody\;level\right)\\ & & =\beta_{0}+\beta_{1}\mathrm{Assessment}+\beta_{2}\mathrm{Assessmen}t^{2}+\beta_{3}\mathrm{Assessmen}t^{3} \end{array}$$

Such a model allows for a smooth and asymmetric transition in vaccine‐induced Ab level regarding assessment time, therefore plausible for rendering the relationship between time and vaccine‐induced Ab levels.

### 4.4 Data preparation

For a comprehensive search, we searched all clinical trial publications related to SARS‐CoV‐2 vaccines from the following databases: EMBASE, Medline, PubMed, Web of Science, WHO Global research database, and medRxiv. All studies published up to September 12, 2021, were searched and selected by three reviewers independently using the following search terms “(COVID‐19 OR SARS‐CoV‐2) AND (vaccine*) AND (clinical trail OR phase trial OR randomized)”. Three independent reviewers judged potentially eligible articles and disagreements were resolved by discussion. The following conditions were required to be included in the study: (1) randomized controlled clinical trials in humans, (2) healthy people who had not been infected with COVID‐19, (3) one type of COVID‐19 vaccine was administered, (4) saline or adjuvant were used as controls, and (5) provides statistical information about the GMT and the 95% CI or about the VE for prevention of COVID‐19; The exclusions were: (1) vaccine of other viruses, (2) report and review, (3) animal trail, (4) unhealthy people, (5) non‐intramuscular injection, (6) not including the schedule used in phase III study, (7) data lacking, (8) duplicate data, and (9) vaccine which lacks data of phase III study or VE.

Three authors were responsible for data extraction independently and discrepancies were resolved through discussion. Characteristics of basic information (author, trial initiation date, trial number, etc.), intervention measures (vaccine type, number of doses, etc.), study design (phase, study type, and age range), assessment method (type of the Ab, evaluation method, and assessment method), Ab information (the GMT and 95% CI each type of Abs), protective efficacy (number of the infected or uninfected participants in the vaccine or placebo group) were extracted. The Ab data in the corresponding study was selected according to the injection dose and injection time of the related phase III studies.

The protective efficacy was defined as the efficacy of the vaccine against the first occurrence of symptomatic COVID‐19. Because of the different evaluation methods for each phase‐III study, the VE was calculated as (1 – RR) × 100, where RR (risk ratio) is the relative risk of incidence rates between the two treatment groups. For vaccines that have more than one study, we use STATA to combine the RR in different groups of studies.

For a single type of vaccine, there could be different schedules and assessment dates, which could therefore lead to bias. To eradicate the discrepancy between experimental designs, only those with the schedules consistent with the phase III experiments were chosen. Besides, the difference in assessment dates was complemented by choosing the date that generated the highest vaccine‐induced Ab level, for different types of vaccines, respectively. The relationship between assessment date and vaccine‐induced Ab levels would also be analyzed later.

The original articles did not necessarily provide raw experimental data. Though it was adequate to use discrete data to determine and rank different types of vaccine‐induced Abs, such statistics failed to crystally reveal the relationship between the Ab levels and efficacy. In order to construct such a linear relationship, Monte Carlo simulation was applied to restore the experimental data. From the original experimental articles, the GMTs and the standard deviations of titer after log_10_ transformation were retrieved for the simulation. We assumed that titer level followed the Gaussian distribution after log_10_ transformation:

$$\mathrm{lo}g_{10}\left(\mathrm{titer}\right)∼N\left(\mu ,\;\sigma^{2}\right)$$

GMT is defined as

$$\mathrm{GMT}\;=\sqrt[{n}]{\prod_{i=1}^{n}\mathrm{tite}r_{i},}$$

which after the log_10_ transformation, we have:

$$\mathrm{lo}g_{10}\;\left(\mathrm{GMT}\right)=\frac{\sum_{i=1}^{n}\mathrm{lo}g_{10}\left(\mathrm{tite}r_{i}\right)}{n},$$

under the assumption of normality after log_10_ transformation, such statistics could be considered as the mean for the Monte Carlo simulation.

Also with the normality assumption, the CIs provided by the original articles with respect to the GMTs could be converted into the estimated standard deviation for log_10_(titer*_i_*):

$$SD_{\mathrm{lo}g_{10}\left(\mathrm{titer}\right)}=\frac{\left(\mathrm{lo}g_{10}\left(\mathrm{UC}I_{\mathrm{GMT}}\right)-\mathrm{lo}g_{10}\left(\mathrm{LC}I_{\mathrm{GMT}}\right)\right)\times \sqrt{n}}{2t_{n\;-\;1,97.5\%}},$$

where UCI_GMT_ stands for the upper bound of the CI for GMT, LCI stands for the lower bound of the CI for GMT, *n* stands for the number of observations, and *t_n_*_– 1,97.5_ stands for the 97.5% quantile of the *t* distribution with *n* – 1 degrees of freedom. We argue that such estimation for the standard deviation of log_10_(titer) is valid as log_10_(GMT) is an unbiased estimator for the mean of log_10_(titer), thus considering that log_10_ transformation preserves the orders, log_10_(UCI_GMT_) and log_10_(LCI_GMT_) are the unbiased estimators for the 97.5% and 2.5% quantile of log_10_(titer), respectively. The more detailed proof for the consistency in quantiles is provided in Supporting Information.

With the assumption of normality, along with the estimated mean and standard error, Monte Carlo Method could be applied to regenerate the data of the original experiments.

Python 3.8.10 was used for data wrangling and validation, R Studio with R version of 4.1.1 was used for simulation, model construction, and interpretation, and ggplot2 was used for visualization and demonstration.

## CONFLICT OF INTEREST

The authors declare that they have no conflict of interest.

## AUTHOR CONTRIBUTIONS

NL, HF, and DP contributed equally to this work. XW, XM and NL designed the study and developed the protocol and analysis plan. NL, HF, DP, and YQ cleaned and analyzed the data. HF, NL contributed to the modeling and statistical analysis of the data. NL, HF, and DP completed the manuscript. All authors contributed the study design and revised the manuscript.

## ETHICS STATEMENT

The manuscript obtained data from original studies, so ethical approval was not required for the study.

## CODE AVAILABILITY

All codes are freely available from https://github.com/COVID‐19‐Vaccine/COVID‐19‐Best‐Immune‐Marker.

## Supporting information



Supporting Information

## Data Availability

All data are freely available from the corresponding author upon request.
