# Supplementary material for: Criteria for judging the immune markers of COVID‐19 disease vaccines
Source: MedComm (2020). 2021 Dec 31;3(1):e109. doi: 10.1002/mco2.109 (PMC8719528; doi:10.1002/mco2.109)
Supplement: Supplementary file 1 — Supporting Information [file MCO2-3-0-s001.pdf]

Criteria for Judging the Immune Markers of COVID-19 Vaccines

Nan Lin[1], Haoxuan Fu[2], Dan Pu3, Yuxin Quan[1], Yueyi Li[4], Xiaomeng Yin[4], Yuhao Wei[1], Hang Wang[1], Xuelei Ma[4], Xiawei Wei[5]

1 West China School of Medicine, West China Hospital, Sichuan University, Chengdu, Sichuan Province, China
2 University of Illinois at Urbana Champaign, Urbana, IL, USA
3 Cancer Center, West China Hospital, Sichuan University, Chengdu, Sichuan Province, China
4 Department of Biotherapy, Cancer Center, West China Hospital, Sichuan University, Chengdu, Sichuan Province, China
5 Laboratory of Aging Research and Nanotoxicology, State Key Laboratory of Biotherapy, National Clinical Research Center for Geriatrics, West China Hospital, Sichuan University, Chengdu, Sichuan Province, China

Correspondence
Xiawei Wei, Laboratory of Aging Research and Nanotoxicology, State Key Laboratory of Biotherapy, National Clinical Research Center for Geriatrics, West China Hospital, Sichuan University.
Email: xiaweiwei@scu.edu.cn
Xuelei Ma, Department of Biotherapy, West China Hospital and State Key Laboratory of Biotherapy, Sichuan University.
Email: drmaxuelei@gmail.com

Table S1: Basic information of included studies.

Table S2: Assessment methods of different vaccines

Table S3: The details of the included studies.

Figure S1: The flowchart for study selection

Supplementary file: Proof for the Consistency in Quantiles

Table S1: Basic information of included studies.

ID	author	Trial Initiation Date	year	trial number	vaccine platform description	type of candidate vaccine	number of doses	schedule	Concentration	phase	num of patients	study type	control	trial country	Age range（year)
1	Keech, C.	2020/5/25	2020	NCT04368988	Protein subunit	SARS-CoV-2 rS/Matrix M1-Adjuvant; NVX-CoV2373	2	Day 0 + 21	5 ug and 25 ug	phase 1/2	131	Randomized, Observer-Blinded, Placebo-controlled	placebo	Australia	18-59
2	Pan, H.	2020/10/7	2021	NCT04758273	Inactivated virus	Inactivated SARS-CoV-2 vaccine (Vero cell)	2	Day 0 + 28	5 ug and 10 ug	phase 1	60	Randomized, Double-blind, Placebo Parallel-controlled	placebo	China	18-59
2	Pan, H.	2020/10/27	2021	NCT04756323	Inactivated virus	Inactivated SARS-CoV-2 vaccine (Vero cell)	2	Day 0 + 28	5 ug and 10 ug	phase 2	500	Randomized, Double-blind, Placebo Parallel-controlled	placebo	China	18-59
3	Xia, S.	2020/4/12	2020	ChiCTR2000031809	Inactivated virus	Inactivated SARS-CoV-2 vaccine (Vero cell)	2	Day 0 + 21	2.5 ug, 5 ug, and 10 μg	phase 1	96	Randomized, Double-blind, Placebo parallel-controlled	Alum	China	18-59
3	Xia, S.	2020/4/12	2020	ChiCTR2000031809	Inactivated virus	Inactivated SARS-CoV-2 vaccine (Vero cell)	2	Day 0 + 21	5 ug	phase 2	224	Randomized, Double-blind, Placebo parallel-controlled	Alum	China	18-59
4	Z. Wu	2020/5/22	2021	NCT04383574	Inactivated virus	CoronaVac; inactivated SARS-CoV-2 vaccine (vero cell)	2	Day 0 + 14	3 ug and 6 ug	phase 1	72	Randomized, Double-blind, Placebo-controlled	placebo	China	≥60
4	Z. Wu	2020/5/22	2021	NCT04383574	Inactivated virus	CoronaVac; inactivated SARS-CoV-2 vaccine (vero cell)	2	Day 0 + 14	1.5 ug, 3 ug and 6 ug	phase 2	350	Randomized, Double-blind, Placebo-controlled	placebo	China	≥60
5	Han, B.	2020/10/31	2021	NCT04551547	Inactivated virus	CoronaVac; inactivated SARS-CoV-2 vaccine (vero cell)	2	Day 0 + 14	1.5 ug and 3.0 ug	phase1	72	Randomized, Double-Blinded, Placebo-Controlled	Alum	China	3-17
5	Han, B.	2020/10/31	2021	NCT04551547	Inactivated virus	CoronaVac; inactivated SARS-CoV-2 vaccine (vero cell)	2	Day 0 + 14	1.5 ug and 3.0 ug	phase 2	480	Randomized, Double-Blinded, Placebo-Controlled	Alum	China	3-17
6	Zhang, Y.	2020/4/16	2021	NCT04352608	Inactivated virus	CoronaVac; inactivated SARS-CoV-2 vaccine (vero cell)	2	Day 0 + 14 or 28	3 ug or 6 ug	phase 1	144	Randomized, Double-blind, Placebo controlled	placebo	China	18-59
6	Zhang, Y.	2020/4/16	2021	NCT04352608	Inactivated virus	CoronaVac; inactivated SARS-CoV-2 vaccine (vero cell)	2	Day 0 + 14 or 28	3 ug or 6 ug	phase 2	600	Randomized, Double-blind, Placebo controlled	placebo	China	18-59
7	Li, M.	2020/5/22	2021	NCT04383574	Inactivated virus	CoronaVac; inactivated SARS-CoV-2 vaccine (vero cell)	2	Day 0 + 14	3 ug and 6 ug	phase 1	72	Randomized, Double-blind, Placebo-controlled	placebo	China	≥60
7	Li, M.	2020/5/22	2021	NCT04383574	Inactivated virus	CoronaVac; inactivated SARS-CoV-2 vaccine (vero cell)	2	Day 0 + 14	1.5 ug, 3 ug and 6 ug	phase 2	350	Randomized, Double-blind, Placebo-controlled	placebo	China	≥60
8	R. Ella	2020/7/15	2021	NCT04471519	Inactivated virus	Whole-Virion Inactivated SARS-CoV-2 Vaccine (BBV152); Covaxin	2	Day 0 + 14	3 ug and 6 ug	phase 1	375	Randomized, Double-blind, Multicenter	Algel	India	18-55
9	R. Ella	2020/7/15	2021	NCT04471519	Inactivated virus	Whole-Virion Inactivated SARS-CoV-2 Vaccine (BBV152); Covaxin	2	Day 0 + 14	3 ug and 6 ug	phase 2	380	Randomized, Double-blind, Multicenter	no	India	12-65
10	Sadoff, J.	2020/7/15	2021	NCT04436276	Viral vector (Non-replicating)	Ad26.COV2.S	1-2	Day 0 or Day 0 +56	$5×10^{10}$ and $1×10^{11}$ viral particles	phase 1/2	805	Randomized, Double-blind, Placebo-controlled	placebo	USA	18-55; ≥65
11	Madhi, S.A.	2020/6/24	2021	NCT04444674	Viral vector (Non-replicating)	ChAdOx1-S - (AZD1222)	1-2	Day 0 + 28	$5×10^{10}$ viral particles	phase 1/2	161	Randomized, Double-blind, Multicenter, Placebo-controlled	placebo	South Africa	18-65
12	Logunov, D. Y.	2020/6/17	2020	NCT04436471	Viral vector (Non-replicating)	Gam-COVID-Vac Adeno-based (rAd26-S+rAd5-S)	2	Day 0 + 21	0.5 ml	phase 1/2	38	Non-randomized, Open, Prospective, Two-stage	no	Russia	18-60
12	Logunov, D. Y.	2020/6/17	2020	NCT04437875	Viral vector (Non-replicating)	Gam-COVID-Vac Adeno-based (rAd26-S+rAd5-S)	2	Day 0 + 21	0.5 ml	phase 1/2	38	Non-randomized, Open, Prospective, Two-stage	no	Russia	18-60
13	Li, J.	2020/7/28	2021	NCT04523571	RNA based vaccine	BNT162b2 (3 LNP-mRNAs), also known as "Comirnaty"	2	Day 0 + 21	10 ug and 20 ug	phase 1	144	Randomized, Observer-blind, Placebo-controlled,	placebo	China	18-55; 65-85
14	Borobia, A. M.	2021/4/24	2021	NCT04860739	RNA based vaccine	BNT162b2 (3 LNP-mRNAs), also known as "Comirnaty"	2	Day 0 + 21	0.3 ml	phase 2	676	Randomized, Multicenter, Open-label, Controlled	placebo	Spain	18-60
15	Jackson, L. A.	2020/3/16	2020	NCT04283461	RNA based vaccine	mRNA-1273	2	Day 0 + 28	25 ug, 100 ug and 250 ug	phase 1	45	Open-Label, Dose-Ranging	no	USA	18-55
16	Anderson, E. J.	2020/3/16	2020	NCT04283461	RNA based vaccine	mRNA-1273	2	Day 0 + 28	25 ug and 100 ug	phase 1	40	Open-Label, Dose-Ranging	no	USA	≥56
17	Chu, L.	2020/5/29	2021	NCT04405076	RNA based vaccine	mRNA-1273	2	Day 0 + 28	50 ug and 100 ug	phase 2	600	Randomized, Observer-Blind, Placebo Controlled, Dose-Confirmation	placebo	USA	≥18
18	Heath. P.T	2020/9/28	2021	EUCTR2020-004123-16-GB	Protein subunit	SARS-CoV-2 rS/Matrix M1-Adjuvant; NVX-CoV2373	2	Day 0 + 21	5 ug	phase 3	14039	Randomized, Observer-blinded, Placebo-controlled	placebo	United Kingdom	18-84
19	Dunkle, L.M.	2020/12/27	2021	NCT04611802	Protein subunit	SARS-CoV-2 rS/Matrix M1-Adjuvant; NVX-CoV2373	2	Day 0 + 21	5 ug/0.5 ml	phase 3	29949	Randomized, Observer-blinded, Placebo-controlled	placebo	USA, Mexico	≥18
20	Al Kaabi	2020/7/16	2021	NCT04510207	Inactivated virus	Inactivated SARS-CoV-2 vaccine (Vero cell)	2	Day 0 + 21	two vaccines with 4 ug or 5ug	phase 3	29582	Randomized, Double-blind, Controlled	alum adjuvant	the United Arab Emirates, Bahrain, Jordan	≥18
21	Tanriover, M.D.	2020/9/14	2021	NCT04582344	Inactivated virus	CoronaVac; inactivated SARS-CoV-2 vaccine (vero cell)	2	Day 0 + 14	3 ug	phase 3	40411	Randomized, Double-blind, Controlled	placebo	Turkey	18-59
22	Fadlyana, E.	2020/8/10	2021	NCT04508075	Inactivated virus	CoronaVac; inactivated SARS-CoV-2 vaccine (vero cell)	2	Day 0 + 14	3 ug/0.5 ml	phase 3	1819	Randomized, Observer-blinded, Placebo-controlled	placebo	Indonesia	18-59
23	R. Ella	2020/11/16	2021	NCT04641481	Inactivated virus	Whole-Virion Inactivated SARS-CoV-2 Vaccine (BBV152); Covaxin	2	Day 0 + 14	6 ug/0.5 ml	phase 3	24419	Randomized, Double-blind, Placebo-controlled	placebo	India	18-98
24	Sadoff, J.	2020/9/7	2021	NCT04505722	Viral vector (Non-replicating)	Ad26.COV2.S	1	Day 0	$5 × 10^{10}$ viral particles	phase 3	44325	Randomized, Double-blind, Placebo controlled	placebo	Argentina, Brazil, Chile, Colombia, Mexico, Peru, South Africa, and the United States	≥18

**25**	Voysey, M.	2020/4/23	2021	NCT04324606	Viral vector (Non-replicating)	ChAdOx1-S - (AZD1222)	1-2	Day 0 + 28	5×10^{10} or 2.2×10^{10} viral particles	phase 1/2	1090	Randomized, Single-blind, Controlled	placebo	UK	18–55
**25**	Voysey, M.	2020/5/28	2021	NCT04400838	Viral vector (Non-replicating)	ChAdOx1-S - (AZD1222)	1-2	Day 0 + 28	5×10^{10} or 2.2×10^{10} viral particles	phase 2/3	7548	Randomized, Single-blind, Controlled	placebo	UK	≥18
**25**	Voysey, M.	2020/6/11	2021	ISRCTN89951424	Viral vector (Non-replicating)	ChAdOx1-S - (AZD1222)	1-2	Day 0 + 28	5×10^{10} or 2.2×10^{10} viral particles	phase 3	4088	Randomized, Single-blind, Controlled	placebo	Brazil	≥18
**25**	Voysey, M.	2020/6/24	2021	NCT04444674	Viral vector (Non-replicating)	ChAdOx1-S - (AZD1222)	1-2	Day 0 + 28	5×10^{10} or 2.2×10^{10} viral particles	phase 1/2	2130	Randomized, Double-blind, Controlled	placebo	South Africa	18-65
**26**	Frater, J.	2020/5/28	2021	NCT04400838	Viral vector (Non-replicating)	ChAdOx1-S - (AZD1222)	1-2	Day 0 + 28	5×10^{10} or 2.2×10^{10} viral particles	phase 2/3	104	Non-randomized, Open-label	MenACWY	UK	18–55
**27**	Emary, K.R.W.	2020/8/3	2021	NCT04400838	Viral vector (Non-replicating)	ChAdOx1-S - (AZD1222)	1-2	Day 0 + 28	5×10^{10} or 2.2×10^{10} viral particles	phase 2/3	8534	Randomized, Single-blind, Controlled	MenACWY	England, Wales, and Scotland	≥18
**28**	Falsey, A.R.	2020/8/28	2021	NCT04516746	Viral vector (Non-replicating)	ChAdOx1-S - (AZD1222)	2	Day 0 + 28	5×10^{10} viral particles	phase 3	32459	Randomized, Double-blind, Placebo-controlled	placebo	USA, Chile, Peru	≥18
**29**	Logunov, D.Y.	2021/5/1	2021	NCT04530396	Viral vector (Non-replicating)	Gam-COVID-Vac Adeno-based (rAd26-S+rAd5-S)	2	Day 0 + 21	10^{11} viral particles	phase 3	21977	Randomized, Double-blind, Controlled	placebo	Moscow, Russia	≥18
**30**	Hall, V.J.	2020/12/7	2021	ISRCTN11041050	RNA based vaccine	BNT162b2 (3 LNP-mRNAs), also known as "Comirnaty"	2	Day 0 + 21	NA	phase 3	23324	Prospective cohort among staff	na	England	≥18
**31**	Polack, F.P.	2020/4/29	2020	NCT04368728	RNA based vaccine	BNT162b2 (3 LNP-mRNAs), also known as "Comirnaty"	2	Day 0 + 21	30 μg	phase 3	43548	Randomized, Observer-blind, Controlled	placebo	United States, Argentina, Brazil, South Africa, Germany, Turkey	≥18
**32**	Baden, L.R.	2020/7/27	2021	NCT04470427	RNA based vaccine	mRNA-1273	2	Day 0 + 28	100 μg	phase 3	30420	Randomized, Observer-blind, Controlled	placebo	United States	≥18
**33**	Frenck, R. W.	2020/4/29	2021	NCT04368728	RNA based vaccine	BNT162b2 (3 LNP-mRNAs), also known as "Comirnaty"	2	Day 0 + 21	30 ug	phase 3	3358	Randomized, Observer-blinded, Placebo-controlled	placebo	United States,	12-25
**34**	Ali, K.	2020/12/9	2021	NCT04649151	RNA based vaccine	mRNA-1273	2	Day 0 + 28	100 ug	phase 3	3732	Randomized, Observer-blinded, Placebo-controlled	placebo	United States,	12-17
**35**	Palacios, R.	2020/7/21	2021	NCT04456595	Inactivated virus	CoronaVac; inactivated SARS-CoV-2 vaccine (vero cell)	2	Day 0 + 14	3 ug/0.5 ml	phase 3	12396	Randomized, Observer-blinded, Placebo-controlled	placebo	Brazil	≥18

1. Keech C, Albert G, Cho I, et al. Phase 1-2 Trial of a SARS-CoV-2 Recombinant Spike Protein Nanoparticle Vaccine. N Engl J Med 2020; 383(24): 2320-32.
2. Pan H, Liu J, Huang B, et al. Immunogenicity and Safety of a SARS-CoV-2 Inactivated Vaccine (KCONVAC) in Healthy Adults: Two Randomized, Double-blind, and Placebo-controlled Phase 1/2 Clinical Trials. medRxiv 2021: 2021.04.07.21253850.
3. Xia S, Duan K, Zhang Y, et al. Effect of an Inactivated Vaccine Against SARS-CoV-2 on Safety and Immunogenicity Outcomes: Interim Analysis of 2 Randomized Clinical Trials. Jama 2020; 324(10): 951-60.
4. Wu Z, Hu Y, Xu M, et al. Safety, tolerability, and immunogenicity of an inactivated SARS-CoV-2 vaccine (CoronaVac) in healthy adults aged 60 years and older: a randomised, double-blind, placebo-controlled, phase 1/2 clinical trial. Lancet Infect Dis 2021; 21(6): 803-12.
5. Han B, Song Y, Li C, et al. Safety, tolerability, and immunogenicity of an inactivated SARS-CoV-2 vaccine (CoronaVac) in healthy children and adolescents: a double-blind, randomised, controlled, phase 1/2 clinical trial. Lancet Infect Dis 2021.
6. Zhang Y, Zeng G, Pan H, et al. Safety, tolerability, and immunogenicity of an inactivated SARS-CoV-2 vaccine in healthy adults aged 18-59 years: a randomised, double-blind, placebo-controlled, phase 1/2 clinical trial. Lancet Infect Dis 2021; 21(2): 181-92.
7. Li M, Yang J, Wang L, et al. A booster dose is immunogenic and will be needed for older adults who have completed two doses vaccination with CoronaVac: a randomised, double-blind, placebo-controlled, phase 1/2 clinical trial. medRxiv 2021: 2021.08.03.21261544.
8. Ella R, Vadrevu KM, Jogdand H, et al. Safety and immunogenicity of an inactivated SARS-CoV-2 vaccine, BBV152: a double-blind, randomised, phase 1 trial. Lancet Infect Dis 2021; 21(5): 637-46.
9. Ella R, Reddy S, Jogdand H, et al. Safety and immunogenicity of an inactivated SARS-CoV-2 vaccine, BBV152: interim results from a double-blind, randomised, multicentre, phase 2 trial, and 3-month follow-up of a double-blind, randomised phase 1 trial. Lancet Infect Dis 2021; 21(7): 950-61.
10. Sadoff J, Le Gars M, Shukarev G, et al. Interim Results of a Phase 1-2a Trial of Ad26.COV2.S Covid-19 Vaccine. N Engl J Med 2021; 384(19): 1824-35.
11. Madhi SA, Baillie V, Cutland CL, et al. Efficacy of the ChAdOx1 nCoV-19 Covid-19 Vaccine against the B.1.351 Variant. N Engl J Med 2021; 384(20): 1885-98.

12. Logunov DY, Dolzhikova IV, Zubkova OV, et al. Safety and immunogenicity of an rAd26 and rAd5 vector-based heterologous prime-boost COVID-19 vaccine in two formulations: two open, non-randomised phase 1/2 studies from Russia. Lancet 2020; 396(10255): 887-97.
13. Li J, Hui A, Zhang X, et al. Safety and immunogenicity of the SARS-CoV-2 BNT162b1 mRNA vaccine in younger and older Chinese adults: a randomized, placebo-controlled, double-blind phase 1 study. Nat Med 2021; 27(6): 1062-70.
14. Borobia AM, Carcas AJ, Pérez-Olmeda M, et al. Immunogenicity and reactogenicity of BNT162b2 booster in ChAdOx1-S-primed participants (CombiVacS): a multicentre, open-label, randomised, controlled, phase 2 trial. Lancet 2021; 398(10295): 121-30.
15. Jackson LA, Anderson EJ, Rouphael NG, et al. An mRNA Vaccine against SARS-CoV-2 - Preliminary Report. N Engl J Med 2020; 383(20): 1920-31.
16. Anderson EJ, Rouphael NG, Widge AT, et al. Safety and Immunogenicity of SARS-CoV-2 mRNA-1273 Vaccine in Older Adults. N Engl J Med 2020; 383(25): 2427-38.
17. Chu L, McPhee R, Huang W, et al. A preliminary report of a randomized controlled phase 2 trial of the safety and immunogenicity of mRNA-1273 SARS-CoV-2 vaccine. Vaccine 2021; 39(20): 2791-9.
18. Heath PT, Galiza EP, Baxter DN, et al. Safety and Efficacy of NVX-CoV2373 Covid-19 Vaccine. N Engl J Med 2021.
19. Dunkle LM, Kotloff KL, Gay CL, et al. Efficacy and Safety of NVX-CoV2373 in Adults in the United States and Mexico. medRxiv 2021: 2021.10.05.21264567.
20. Al Kaabi N, Zhang Y, Xia S, et al. Effect of 2 Inactivated SARS-CoV-2 Vaccines on Symptomatic COVID-19 Infection in Adults: A Randomized Clinical Trial. Jama 2021; 326(1): 35-45.
21. Tanriover MD, Doğanay HL, Akova M, et al. Efficacy and safety of an inactivated whole-virion SARS-CoV-2 vaccine (CoronaVac): interim results of a double-blind, randomised, placebo-controlled, phase 3 trial in Turkey. Lancet 2021; 398(10296): 213-22.
22. Fadlyana E, Rusmil K, Tarigan R, et al. A phase III, observer-blind, randomized, placebo-controlled study of the efficacy, safety, and immunogenicity of SARS-CoV-2 inactivated vaccine in healthy adults aged 18–59 years: An interim analysis in Indonesia. Vaccine 2021.
23. Ella R, Reddy S, Blackwelder W, et al. Efficacy, safety, and lot to lot immunogenicity of an inactivated SARS-CoV-2 vaccine (BBV152): a, double-blind, randomised, controlled phase 3 trial. medRxiv 2021: 2021.06.30.21259439.
24. Sadoff J, Gray G, Vandebosch A, et al. Safety and Efficacy of Single-Dose Ad26.COV2.S Vaccine against Covid-19. N Engl J Med 2021; 384(23): 2187-201.
25. Voysey M, Clemens SAC, Madhi SA, et al. Safety and efficacy of the ChAdOx1 nCoV-19 vaccine (AZD1222) against SARS-CoV-2: an interim analysis of four randomised controlled trials in Brazil, South Africa, and the UK. Lancet 2021; 397(10269): 99-111.
26. Frater J, Ewer KJ, Ogbe A, et al. Safety and immunogenicity of the ChAdOx1 nCoV-19 (AZD1222) vaccine against SARS-CoV-2 in HIV infection: a single-arm substudy of a phase 2/3 clinical trial. Lancet HIV 2021.
27. Emary KRW, Golubchik T, Aley PK, et al. Efficacy of ChAdOx1 nCoV-19 (AZD1222) vaccine against SARS-CoV-2 variant of concern 202012/01 (B.1.1.7): an exploratory analysis of a randomised controlled trial. Lancet 2021; 397(10282): 1351-62.
28. Falsey AR, Sobieszczyk ME, Hirsch I, et al. Phase 3 Safety and Efficacy of AZD1222 (ChAdOx1 nCoV-19) Covid-19 Vaccine. New England Journal of Medicine 2021.
29. Logunov DY, Dolzhikova IV, Shcheblyakov DV, et al. Safety and efficacy of an rAd26 and rAd5 vector-based heterologous prime-boost COVID-19 vaccine: an interim analysis of a randomised controlled phase 3 trial in Russia. Lancet 2021; 397(10275): 671-81.
30. Hall VJ, Foulkes S, Saei A, et al. COVID-19 vaccine coverage in health-care workers in England and effectiveness of BNT162b2 mRNA vaccine against infection (SIREN): a prospective, multicentre, cohort study. Lancet 2021; 397(10286): 1725-35.
31. Polack FP, Thomas SJ, Kitchin N, et al. Safety and Efficacy of the BNT162b2 mRNA Covid-19 Vaccine. N Engl J Med 2020; 383(27): 2603-15.
32. Baden LR, El Sahly HM, Essink B, et al. Efficacy and Safety of the mRNA-1273 SARS-CoV-2 Vaccine. N Engl J Med 2021; 384(5): 403-16.
33. Frenck RW, Jr., Klein NP, Kitchin N, et al. Safety, Immunogenicity, and Efficacy of the BNT162b2 Covid-19 Vaccine in Adolescents. N Engl J Med 2021; 385(3): 239-50.

34. Ali K, Berman G, Zhou H, et al. Evaluation of mRNA-1273 SARS-CoV-2 Vaccine in Adolescents. N Engl J Med 2021.
35. Palacios R, Batista AP, Albuquerque CSN, et al. Efficacy and Safety of a COVID-19 Inactivated Vaccine in Healthcare Professionals in Brazil: The PROFISCOV Study. SSRN; 2021.

Table S2: Assessment methods of different vaccines

Vaccine platform description	Type of candidate vaccine	ID	Author	Subgroups	Type of antibody	Evaluation method	Details of the method	schedule	Assessment time (Day X)									
									0	7	14	21	28	35	42	56	70	other
Protein subunit	NVX-CoV2373	1	Keech, C.	5 ug	IgG responses to rSARS-CoV-2	ELISA		Day 0 + 21	√	√		√	√	√				
					neutralizing Ab to live SARS-Cov-22	MN	IC>99%		√			√		√				
Inactivated virus	BIBP-CorV	2	Pan, H.	5 ug	neutralizing Ab to live SARS-Cov-2	CPE	a detection limit of 1:4	Day 0 + 14	√				√		√			
					neutralizing antibody to pseudovirus	pseudovirus neutralization tests	a detection limit of 1:10		√				√		√			
					RBD-IgG	ELISA	a detection limit of 1:20		√				√		√			

		2	Pan, H.	5 ug	neutralizing Ab to live SARS-Cov-2	CPE	a detection limit of 1:4	Day 0 + 14	√				√		√			
								Day 0 + 28	√						√	√		
					neutralizing antibody to pseudovirus	pseudovirus neutralization tests	a detection limit of 1:10	Day 0 + 14	√				√		√			
								Day 0 + 28	√						√	√		
					RBD-IgG	ELISA	a detection limit of 1:20	Day 0 + 14	√				√		√			

								Day 0 + 28	√						√	√		
		3	Xia, S.	Medium dose	neutralizing Ab to live SARS-Cov-2	$PRNT_{50}$		Day 0 + 28 + 56	√		√	√					√	
		3	Xia, S.	Medium dose	binding Ab	ELISA*	coating antigen: the inactivated whole SARS-CoV-2	Day 0 + 28 + 56	√		√	√					√	
		3	Xia, S.	Medium dose	neutralizing Ab to live SARS-Cov-2	$PRNT_{50}$		Day 0 + 14	√				√					
		3	Xia, S.	Medium dose	neutralizing Ab to live SARS-Cov-2	$PRNT_{50}$		Day 0 + 21	√					√				

						ELISA*	coating antigen: the inactivated whole SARS-CoV-2	Day 0 + 14	√				√					
								Day 0 + 21	√					√				
		20	Al Kaabi	5 ug	neutralizing Ab to live SARS-Cov-2	ELISA		Day 0 + 21	√					√				
	Corona Vac	4	Z. Wu	3 ug	neutralizing Ab to live SARS-Cov-2	CPE		Day 0 + 28	√				√			√		
		4	Z. Wu	3 ug	neutralizing Ab to live SARS-Cov-2	CPE		Day 0 + 28	√							√		

		5	Han, B.	3 ug	neutralizing Ab to live SARS-Cov-2	CPE		Day 0 + 28	√				√			√		
		5	Han, B.	3 ug	neutralizing Ab to live SARS-Cov-2	CPE		Day 0 + 28	√							√		
		6	Zhang , Y.	3 ug	neutralizing Ab to live SARS-Cov-2	CPE	a minimum 4-fold dilution	Day 0 + 14	√	√	√	√	√		√			
								Day 0 + 28	√				√	√	√	√		
					RBD-IgG	ELISA*	from Sinovac, a minimum 160-fold dilution	Day 0 + 14	√	√	√	√	√		√			

								Day 0 + 28	√				√	√	√	√		
					neutralizing antibodies to pseudovirus	CPE	a minimum 10-fold dilution	Day 0 + 14		√	√	√	√					
		6	Zhang , Y.	3 ug	neutralizing Ab to live SARS-Cov-2	CPE	a minimum 4-fold dilution	Day 0 + 14	√			√	√		√			
								Day 0 + 28	√							√		
					RBD-IgG	ELISA*	from Sinovac, a minimum 160-fold dilution	Day 0 + 14	√			√	√		√			

								Day 0 + 28	√							√		
					neutralizing antibodies to pseudovirus	CPE	a minimum 10-fold dilution	Day 0 + 14					√					
		7	Li, M.	3 ug	neutralizing Ab to live SARS-Cov-2 Ab to live SARS-Cov-2	CPE		Day 0 + 28	√			√	√		√			208
		7	Li, M.	3 ug	neutralizing Ab to live SARS-Cov-2	CPE		Day 0 + 28	√							√		208
		22	Fadlya na, E.	3 ug	neutralizing Ab to live SARS-Cov-2	NAb		Day 0 + 14	√				√					

					RBD-IgG	ELISA			√				√					
	BBV152	8	R. Ella	6 µg with Algel – IMDG	neutralizing Ab to live SARS-Cov-2	MN_{50}		Day 0 + 14	√		√		√		√			104
					RBD-IgG	ELISA*			√				√					
					Ab to spike glycoprotein	ELISA*			√				√					
					N-IgG	ELISA*			√				√					

		9	R. Ella	6 µg with Algel – IMDG	neutralizing Ab to live SARS-Cov-2	MN_{50}*		Day 0 + 14	√				√		√	√		
						$PRNT_{50}$*			√				√		√	√		
					RBD-IgG	ELISA			√				√		√	√		
					Ab to spike glycoprotein	ELISA			√				√		√	√		
					N-IgG	ELISA			√				√		√	√		

		23	R. Ella	6 µg with Algel – IMDG	neutralizing Ab to live SARS-Cov-2	MN$_{50}$		Day 0 + 14	√							√		
					RBD-IgG	ELISA										√		
					Ab to spike glycoprotein	ELISA										√		
					N-IgG	ELISA										√		
Viral vector (Non-replicating)	Ad26.COV2.S	10	Sadoff, J.	Low Dose/Placebo; 18-55years	neutralizing Ab to live SARS-Cov-2	MN		Day 0	√				√			√		

				Low Dose; ≥65 years	neutralizing Ab to live SARS-Cov-2	MN			√		√		√					
		11	Madhi, S.A.	HIV-negative	neutralizing Ab to live SARS-Cov-2	MN	Asp614Gly wild-type, ID_{50}	Day 0 + 28	√				√		√			
	ChAdOx1-S - (AZD1222)	26	Frater, J.	patient HIV negative	Ab to spike glycoprotein	ELISA		Day 0 + 28	√		√		√		√	√		
		27	Emary, K.R.W.	all	Ab to spike glycoprotein	ELISA		Day 0 + 28	√		√		√			√		
	Gam-COVID-Vac	12	Logunov, D. Y.	rAd26-S + rAd5-S; Gam-COVID-Vac	neutralizing Ab to live SARS-Cov-2	MN	$TCID_{50}$	Day 0 + 21	√		√		√		√			

					RBD-IgG	ELISA			√		√	√	√		√			
		12	Logunov, D. Y.	rAd26-S + rAd5-S; Gam-COVID-Vac-Lyo	neutralizing Ab to live SARS-Cov-2	MN	TCID$_{50}$	Day 0 + 21	√		√		√		√			
					RBD-IgG	ELISA			√		√	√	√		√			
		29	Logunov, D. Y.	1×10^{11} viral particles vaccine	neutralizing Ab to live SARS-Cov-2	MN	TCID$_{50}$	Day 0 + 21							√			
					RBD-IgG	ELISA									√			

RNA based vaccine	BNT16 2b2	13	Li, J.	30 ug + BNT16 2b 1; 18-55 years	neutralizing Ab to live SARS-Cov-2	CPE		Day 0 + 21	√	√		√	√		√			
					RBD-IgG	ELISA			√	√		√	√		√			
					S1 binding Ab	ELISA			√	√		√	√		√			
				30 ug + BNT16 2b 1; 65-85 years	neutralizing Ab to live SARS-Cov-2	CPE			√			√	√		√			
					RBD-IgG	ELISA			√			√	√		√			

					S1 binding Ab	ELISA			√			√	√		√			
		14	Borobia, A. M.	Interventional group	RBD-IgG	The Elecsys Anti SARS-CoV-2 S assay	a electrochemiluminescence immunoassay	Day 0	√	√	√							
					Ab to spike glycoprotein	TrimericS IgG assay	a chemiluminescence immunoassay		√	√	√							
					neutralizing antibodies to pseudovirus	pseudovirus neutralization assay	NT_{50}		√		√							
		34	Frenck R.W.	30 ug	neutralizing Ab to live SARS-Cov-2	SARS-CoV-2 serum neutralization assay	NT_{50}	Day 0 + 21	√									51

	mRNA-1273	15	Jackson, L. A.	100 ug	RBD-IgG	ELISA		Day 0 + 28	√		√		√	√	√	√		
					Binding antibody IgG to S-2P	ELISA			√		√		√	√	√	√		
					neutralizing Ab to live SARS-Cov-2	PRNT80$_{80}$			√						√			
					neutralizing antibodies to pseudovirus	PsVNA	ID$_{50}$		√		√		√	√	√	√		
		16	Anderson, E. J.	100 ug	RBD-IgG	ELISA		Day 0 + 28	√		√		√	√	√	√		

					Binding antibody IgG to S-2P	ELISA			√		√		√	√	√	√		
					neutralizing antibodies to pseudovirus	PsVNA	against the 614D variant		√		√		√	√	√	√		
							against the 614G/Gly polymorphic variant								√			
					neutralizing Ab to live SARS-Cov-2	nLuc HTNA			√				√		√			
						FRNT mNG			√				√		√			

						PRNT			√						√			
		17	Chu, L.	100 ug	Binding antibody IgG to S-2P	ELISA		Day 0 + 28	√				√		√	√		
					neutralizing Ab to live SARS-Cov-2	MN	based on an in situ ELISA readout		√				√		√	√		
		33	Ali. K	100 ug	neutralizing antibodies to pseudovirus	Pseudovirus neutralization assay	Wuhan-Hu-1 isolate including D614G	Day 0 + 28							√			
					binding Ab	ELISA									√			

* means the assay is in-house developed.

ELISA: enzyme-linked immunosorbent assay.

MN: a live virus micro neutralization assay.

IC: inhibitory concentration.

PRNT: SARS-CoV-2 plaque-reduction neutralization testing assay, which uses wild-type virus; $PRNT_{50}$ OR $PRNT_{80}$ values were reported as a measure to determine the extent to which

serum can be diluted and still reduce SARS-CoV-2 plaque formation by 50% OR 80%.

CPE: live SARS-CoV-2 micro cytopathogenic effect assay.

Nab: the neutralization of antibody assay.

ID_{50}: inhibitory dilution 50%.

$TCID_{50}$: a 50% tissue culture infective dose of 100.

NT_{50}: The titer of neutralizing antibodies was calculated as 50% inhibitory dose.

PsVNA: a pseudotyped lentivirus reporter single-round-of-infection neutralization assay.

nLuc HTNA: the SARS-CoV-2 nanoluciferase high throughput neutralization assay, which uses a virus expressing the reporter gene nanoluciferase (nLuc).

FRNT mNG: the focus reduction neutralization test mNeonGreen, which uses recombinant SARS-CoV-2 expressing the fluorescent reporter gene mNeon-Green

Table S3: The details of the included studies.

Reference	vaccine platform	vaccine type	Dosage	Measure of effectiveness	vaccine efficacy*
24	Viral vector (Non-replicating)	Ad26.COV2.S	5 × 10^{10} viral particles	Symptomatic Covid-19 of any severity	66.6%
31	RNA based vaccine	BNT162b2	30 µg	Covid-19 occurrence at least 7 days after the second dose	95.1%
25, 27, 28	Viral vector (Non-replicating)	ChAdOx1-S - (AZD1222)	5 × 10^{10} viral particles	Covid-19 occurrence at least 14 days after the second dose	67.9%
21, 22, 35	Inactivated virus	CoronaVac	3 ug/0.5 ml	Symptomatic COVID-19 with onset at least 14 days after the second injection	56.8%
29	Viral vector (Non-replicating)	Gam-COVID-Vac	10^{11} viral particles	First COVID-19 occurrence from 21 days after dose 1 (day of dose 2)	91.6%
20	Inactivated virus	BIBP-CorV	5 ug/0.5 mg and 4 ug/0.45 mg	Symptomatic Covid-19 occurrence at least 14 days after the second dose	75.1%
32, 34	RNA based vaccine	mRNA-1273	100 µg	Covid-19 occurrence at least 14 days after the second dose	95.1%
18, 19	Protein subunit	NVX-CoV2373	5 ug/0.5 ml	Covid-19 occurrence at least 7 days after the second dose	89.2%
23	Inactivated virus	BBV152	6 ug/0.5 ml	Covid-19 occurrence at least 14 days after the second dose	77.5%

*The vaccine efficacy was calculated using the original data from the phase III studies and combined using Stata.

Figure S1: The flowchart for study selection

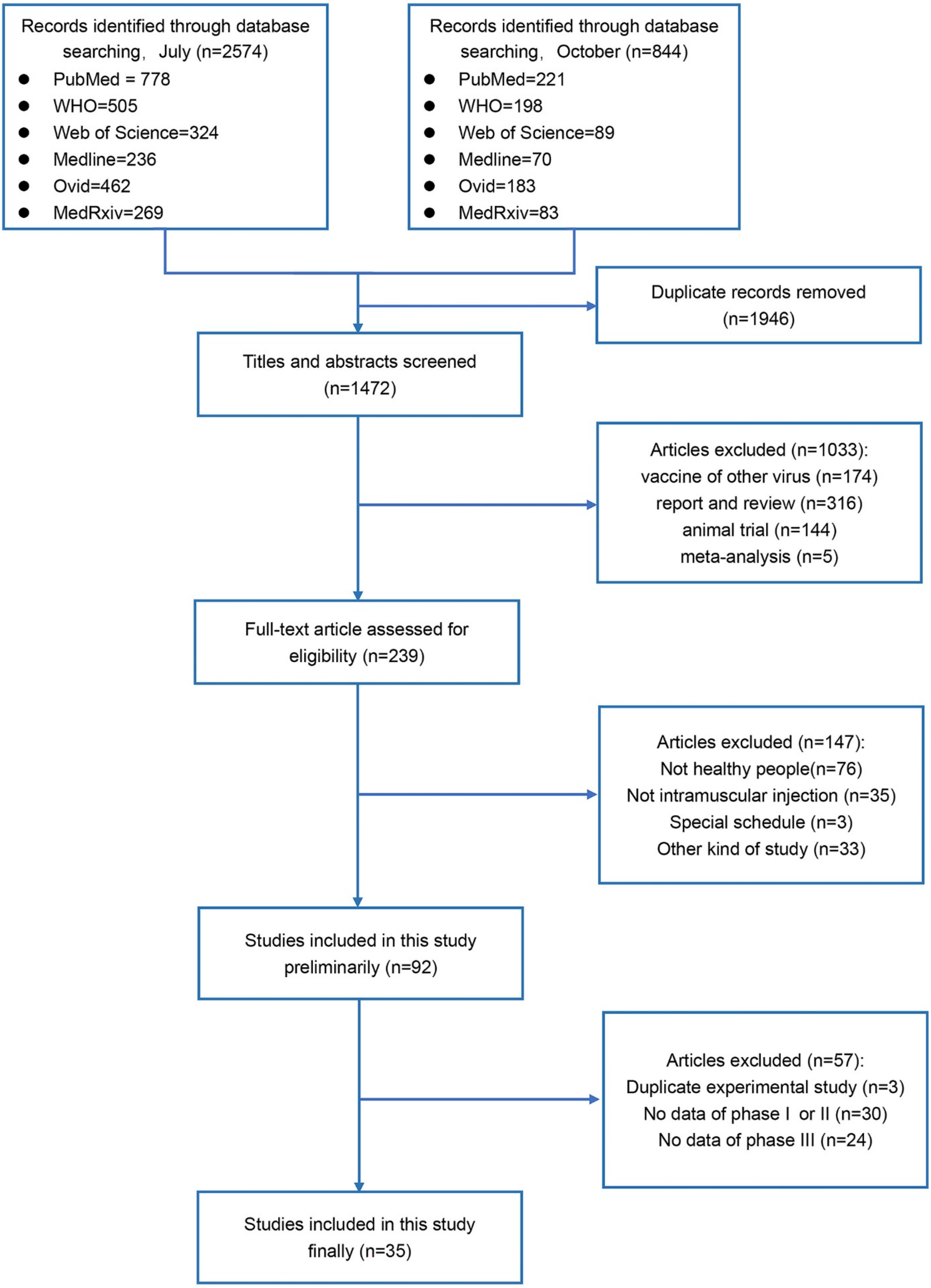

Supplementary file: Proof for the Consistency in Quantiles

This proof is for verifying the plausibility of simply taking the log_{10} transformation over the quantile of GMT would lead to the the quantile of it in $\frac{\sum_{i=1}^{n} log_{10}(titer_i)}{n}$.

GMT is defined as:

$$\text{Geometric Mean Titers} = \sqrt[n]{\prod_{i=1}^{n} titer_i}. \tag{1}$$

Under the normality assumption of titer, the consistency of quantiles regarding GMT and $\frac{\sum_{i=1}^{n} log_{10}(titer_i)}{n}$ can be verified by the following proof:

$$\frac{\sum_{i=1}^{n} log_{10}(titer_i)}{n} \sim \mathcal{N}(\mu, \frac{\sigma^2}{n}), \tag{2}$$

therefore its probability density function can be explicitly written as:

$$f(x_s) = \frac{1}{\sqrt{2\pi}\frac{\sigma}{\sqrt{n}}} e^{-\frac{1}{2}(\frac{x-\mu}{\frac{\sigma}{\sqrt{n}}})^2}, \tag{3}$$

where x represents the arithematic mean of $log_{10}(titer)$ of a specific type of vaccine. The probability density function of $\sqrt[n]{\prod_{i=1}^{n} titer_i}$ can therefore be written as:

$$g(y_s) = \frac{1}{ln(10)\sqrt{2\pi}\frac{\sigma}{\sqrt{n}}y} e^{-\frac{1}{2}(\frac{log_{10}(y)-\mu}{\frac{\sigma}{\sqrt{n}}})^2}, \tag{4}$$

where y_s represents the geometric mean of titers of a specific type of vaccine.

Assume that $X_{0.975}$ represents the 97.5_{th} quantile of $\frac{\sum_{i=1}^{n} log_{10}(titer_i)}{n}$, then by the definition of quantile, we have:

$$\int_{-\infty}^{X_{0.975}} \frac{1}{\sqrt{2\pi}\frac{\sigma}{\sqrt{n}}} e^{-\frac{1}{2}(\frac{x-\mu}{\frac{\sigma}{\sqrt{n}}})^2} dx = 0.975, \tag{5}$$

replacing x with $log_{10}(y)$, we have:

$$\int_{0}^{10^{X_{0.975}}} \frac{1}{ln(10)\sqrt{2\pi}\frac{\sigma}{\sqrt{n}}y} e^{-\frac{1}{2}(\frac{log_{10}(y)-\mu}{\frac{\sigma}{\sqrt{n}}})^2} dy = 0.975, \tag{6}$$

Notice that the integrand is just the probability density function of $\sqrt[n]{\prod_{i=1}^{n} titer_i}$, thus $10^{X_{0.975}}$ is the 97.5_{th} quantile for $\sqrt[n]{\prod_{i=1}^{n} titer_i}$.
